# Supplementary material for: The most complete enantiornithine from North America and a phylogenetic analysis of the Avisauridae
Source: PeerJ. 2018 Nov 13;6:e5910. doi: 10.7717/peerj.5910 (PMC6238772; doi:10.7717/peerj.5910)
Supplement: Supplemental Information 1 [file peerj-06-5910-s001.docx]

Supplemental Information

Modified Character 233 (state 3 new)

Metatarsal II tubercle (associated with the insertion of the tendon of the m. tibialis cranialis in Aves): absent (0); present, on the medial dorsomedial margin of metatarsal II (1); located approximately the center of the proximodorsal surface of metatarsal II (2); developed on lateral surface of metatarsal II, at contact with metatarsal III or on lateral edge of metatarsal III (3). (ORDERED)

New Characters 246 – 252:

246. Metatarsals II and III, tubercle for muscle attachment (presumably the m. tibialis cranialis): one such tubercle (0); two tubercles present (1). (modified from O’Connor et al., 2014 – *Evgenavis*).

247. Position of the tubercle for the m. tibialis cranialis: proximally located (0); located approximately 1/3 from the proximal end (1); located near to or distal from the midpoint (2). (from O’Connor, 2009)

248. Tubercle for the m. tibialis cranialis hypertrophied: absent (0); present (1). (New)

249. Medial condyle of metatarsal III trochlea projecting strongly plantarly relative to the lateral condyle: absent (0); present (1); lateral condyle projects farther (2). (modified from Chiappe, 1993).

250. Medial trochlea of metatarsal III, protrudes farther distally than lateral trochlea: absent (0); present (1). (New)

251. Dorsal surface of metatarsal III strongly convex: absent (0); present (1). (from Chiappe, 1993)

252. Metatarsal IV trochlea: ginglymous (0); reduced to a single convex surface in caudal view (1); single condyle medially excavated (2). (modified from Chiappe, 1993).

Synapomorphies:

Avisauridae

107: 2 -> 1 (reduction in hypocleidium, only Neuquenornis)

127: 0 -> 1 (well developed pneumatic fossa on proximal humerus)

249: 0 -> 1 medial condyle of metatarsal III trochlea with strong plantar projection (Chiappe 1993)

NAM Avisaurids:

248: 0 -> 1 hypertrophied tubercle for the m. tibialis cranialis

252: 1 -> 2 metatarsal IV trochlea medially excavated

SAM Avisaurids:

217: 1 -> 0 gradual sloping of tibiotarsal condyles towards midline of the tibiotarsus

226: 0 -> 1 proximal vascular foramen between metatarsals III and IV

Mystiornis polytomy + Avisauridae:

98: 0 -> 1 costolaterally wide acromion process on scapula

110: 1 -> 0 keel reaching rostral margin sternum

111: 2 -> 1 single pair of sternal trabeculae

238: 0 -> 1 trochlea of metatarsal II broader than that of metatarsal III

Gettyia gloriae:

231: 1 – distal foramen completely enclosed by metatarsals III and IV

Mirarce

223: 0 – reversal, proximal tarsometatarsus not dorsally inclined

Mirarce + Gettyia:

230: 1 -> 0 plantar tarsometatarsus not excavated

247: 1 -> 2 tubercle for the m. tibialis cranialis located near the midpoint of the tarsometatarsus
